# Predictors of first-year statin medication discontinuation: A cohort study

**DOI:** 10.1016/j.jacl.2016.04.010

**Published:** 2016

**Authors:** Heli Halava, Risto Huupponen, Jaana Pentti, Mika Kivimäki, Jussi Vahtera

**Affiliations:** a Department of Public Health, University of Turku, Turku, Finland; b Department of Pharmacology, Drug Development and Therapeutics, University of Turku, Turku, Finland; c Tykslab, Turku University Hospital, Turku, Finland; d Finnish Institute of Occupational Health, Turku, Finland; e Department of Epidemiology and Public Health, University College of London, London, UK; f Department of Public Health, Clinicum, Faculty of Medicine, University of Helsinki, Helsinki, Finland; g Turku University Hospital, Turku, Finland

**Keywords:** Discontinuation, Statins, Adherence

## Abstract

**Background:**

The discontinuation of statin medication is associated with an increased risk of cardiovascular and cerebrovascular events and, among high-risk patients, all-cause mortality, but the reasons for discontinuation among statin initiators in clinical practice are poorly understood.

**Objective:**

To examine factors predicting the early discontinuation of statin therapy.

**Methods:**

In this prospective cohort study, participants with baseline measurements before the initiation of statin treatment were linked to national registers and followed for the discontinuation of statins during the first year of treatment (no filled prescriptions after statin initiation within the subsequent 12 months).

**Results:**

Of all the 9285 statin initiators, 12% (n = 1142) were discontinuers. Obesity, overweight, vascular comorbidities, and older age were independently associated with a reduced risk of discontinuation [odds ratios (OR) = 0.82 (95% confidence interval [CI], 0.69–0.99), 0.85 (95% CI, 0.73–0.98), 0.80 (95% CI, 0.68–0.93), and 0.82 (95% CI, 0.68–0.99), respectively]. In contrast, high-patient cost-sharing was associated with an increased odds (OR = 1.29; 95% CI, 1.03–1.62) for discontinuation. The only significant difference between the sexes (*P* = .002) was observed among the participants with risky alcohol use, which was associated with a decreased odds for discontinuation among the men (OR = 0.69; 95% CI, 0.49–0.98) and an increased odds among the women (OR = 1.28; 95% CI, 1.02–1.62).

**Conclusions:**

The discontinuation of statin therapy during the first year after initiation is common. Lowering out-of-pocket expenditures and focusing on low-risk patient groups and women with risky alcohol use could help maintain the continuation of medication.

## Highlights

• Of the 9285 statin initiators, 12% discontinued treatment within the first year.
• High-patient co-payment was an independent risk factor for the discontinuation.
• The only significant difference between genders was observed for risky alcohol use.
• Risky alcohol use predicted discontinuation of treatment in women but not in men.

**What is already known about this subject?**
Despite well-documented benefits of statins, discontinuation of statin medication is common among primary and secondary prevention patients.

**What does this study add?**
Several predictors of discontinuation are readily assessable and can provide information with which to identify those with an increased risk of nonadherence.

**How might this impact on clinical practice?**
In clinical practice, many patients for whom statins are prescribed discontinue the use of the drug within a year, which is likely to reduce any benefit of medication and increase the risk of cardiovascular events. Increased efforts to motivate treatment adherence in risk groups could reduce discontinuation and cardiovascular events.

## Introduction

Statins are one of the most widely studied and evidence-based medications^1^ and are an essential component of cardiovascular disease prevention. Statins are well tolerated, safe, and inexpensive (when following the generic substitution). Despite these well-documented benefits, poor adherence to statins and an extreme form of it, discontinuation—that is, quitting statin medication use^2^—is common among primary and secondary prevention patients.^3^, ^4^, ^5^, ^6^ In clinical trials, the discontinuation rates range from 4% to 11%,^7^, ^8^, ^9^ but in routine care, the rates are much higher; between 11% and 53%.^2^, ^3^, ^10^, ^11^ According to studies using electronic medical records, approximately 25% to 50% of patients discontinue statin use within six months to one year after initiating their use.^4^, ^5^, ^12^ The number of patients continuing therapy falls sharply in the first few months of treatment, followed by a more gradual decline.^4^, ^13^

Discontinuation is commonly attributed to statin-related adverse events, but, because most patients who reinitiate statin use can tolerate this medication long-term,^2^ many of these events may have had other etiologies. Previous studies on the determinants of discontinuation have reported mixed results. Some found a greater tendency to discontinue statin treatment among the young (<50 years) or old (>70 years),^11^, ^14^, ^15^, ^16^ with high co-payment,^2^, ^3^, ^17^, ^18^ for primary prevention patients,^3^, ^5^, ^15^, ^19^, ^20^, ^21^ and intensive dose therapy.^22^ An increased risk of statin discontinuation has also been found for smokers,^20^ and patients with diabetes^20^, ^23^ despite the current guidelines recommending statin medication for nearly all patients with type 2 diabetes.^24^ On the contrary, one study found diabetes to be associated with the continuation of lipid-lowering drugs (of which statins accounted for 69%).^25^ However, other studies have shown no association between discontinuation and age,^19^, ^20^, ^26^, ^27^, ^28^, ^29^ diabetes,^6^, ^16^, ^27^, ^29^ or smoking.^27^

This study was aimed at identifying patient groups with an increased likelihood to discontinue statins in a large prospective cohort linked to prescription registers. A better understanding of the determinants of adherence to statin treatment is important because discontinuation is common, and it significantly increases both the incidence of cardiovascular and cerebrovascular events^30^ and, among high-risk patients, also all-cause mortality.^31^, ^32^ Because the decision concerning the continuation of statin-use is commonly made during the first year of treatment,^3^, ^33^ we restricted our analysis to predictors of discontinuation during the first year of medication.

## Methods

### Study population and design

The data used in this study came from the Finnish Public Sector Study,^34^ a prospective study of all local government employees of 10 towns and all employees in 21 public hospitals with a ≥6-month job contract in 1991–2005.

**Figure 1:**
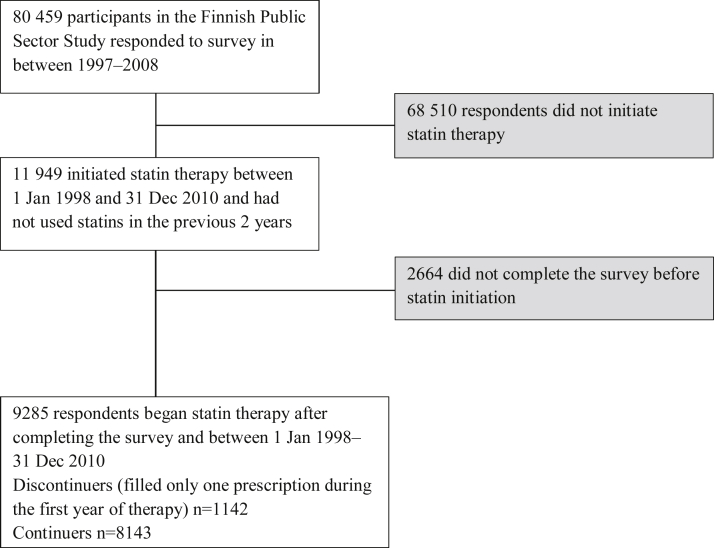
Flow chart of sample selection.

We initially included the 80,459 identifiable participants who responded to a survey in 1997–1998, 2000–2002, 2004, or 2008. The questionnaires involved demographic characteristics, lifestyle factors, and health status, and the average response rate was 70%. We linked the survey data to data from national health registers using unique personal identification numbers as in our earlier studies.^34^, ^35^ Among the respondents, there were 11,949 participants who had initiated statin medication between 1 January, 1998 and 31 December, 2010. Of them, we included all the 9285 participants who had completed a survey before the statin therapy began and had not been dispensed statins in the previous two years. From the initiators, we identified the 1142 discontinuers (initiators who filled only one prescription during the first year of statin treatment; Figure 1). Follow-up data were available until 31 December 2011. In cases of repeated surveys before initiation, we selected the most recent response. The mean lag between the response and statin initiation was 3.4 years (standard deviation, SD ± 2.4).

### Discontinuation of statin treatment

In Finland, statins are available by prescription only. The National Health Insurance Scheme provides prescription drug coverage for all (∼5.4 million) community-dwelling residents. All reimbursed prescriptions are registered in the Finnish Prescription Register managed by The Social Insurance Institution of Finland.^36^ Reimbursed medicines can be supplied to a patient for three months per purchase. For each drug, reimbursement-related factors including the dispensing date, the World Health Organization Anatomical Therapeutic Chemical (ATC) code,^37^ the quantity dispensed, and co-payment are recorded.

From this register, we identified all statin users, based on filled prescriptions with the ATC code C10AA. All the patients who initiated statin therapy were assumed to require treatment for the rest of their lives. Discontinuation of statin therapy was considered to take place when after the first filled prescription, no more statin prescriptions were filled within the subsequent 12 months. We also recorded co-payment and the year of statin initiation due to major changes in prescribing practices and statin costs over time.^38^

### Independent covariates

We assessed lifestyle factors using standard questionnaire measurements.^34^, ^35^ We requested the participants' smoking status (none, former, current) and calculated body mass index using self-reported weight and height. We classified body mass index into the following three groups: normal weight (<25 kg/m^2^), overweight (25–29.9 kg/m^2^), and obese (≥30 kg/m^2^). We defined risky alcohol user as a participant with either a high mean alcohol consumption (≥16 drinks per week for women and ≥24 per week for men, one unit equal to 12 cl of wine or 4 cl of spirits or 33 cl of beer) or having passed out due to heavy alcohol consumption at least once during the 12 months or both. Physical activity was measured by the Metabolic Equivalent Task index; the sum score of the Metabolic Equivalent Task hours was used to identify active (>4 hours), moderate (2–4 hours), or low (<2 hours) physical activity.

We identified cardiovascular comorbidities (including cardiovascular diseases and diabetes) using special reimbursement and hospital discharge registers (entitlements to special reimbursement for drug treatment of chronic hypertension, heart failure, coronary artery disease, or diabetes at statin initiation, or hospitalization for these conditions, stroke, or arrhythmias during 36 months before initiation).^34^, ^35^ Information on cancer diagnosis within five years before statin initiation came from the Finnish Cancer Registry.^39^ Antidepressant purchases (ATC code N06 A) during the 36 months preceding statin initiation, captured from the Prescription Register and served as a proxy for depression. Information on self-rated health (classified as suboptimal if average or worse vs not if good or very good) and marital status (married or cohabiting vs single, divorced or widowed) were obtained from the survey responses. Data on sex and age (24–50, 51–60, 61–75 years) came from the employers' administrative registers. Information on co-payment per first statin purchase (<5 euros, 5–20 euros, >20 euros) came from the Finnish Prescription Register. Statistics Finland provided information on education, which was classified as high (tertiary level), intermediate (upper secondary level), or basic (lower secondary level or less).^40^

### Statistical analyses

We used a logistic regression analysis to estimate the association of discontinuation with demographic characteristics, comorbidities, lifestyle factors, and co-payment. Only the respondents with complete data on all the predictors were included. The first model was adjusted for the year of statin initiation. All the found significant predictors of statin discontinuation were then simultaneously entered into the second model to examine their independent effects on discontinuation. The data were analyzed with SAS software, version 9.2 (SAS Institute, Inc., Cary, NC).

### Ethics approval

The study was approved by the ethics committee of the Hospital District of Helsinki and Uusimaa.

## Results

### Study population characteristics

The participants were predominately women (76%) and highly educated (47%), and they were aged 55.7 years on average. Almost one third of them had vascular comorbidity, and one fifth was obese. Behavioral health risks were common: 31% were physically inactive, 17% were current smokers, and 14% were risky alcohol users (Table 1).

### Discontinuation

Of the 9285 statin initiators, 88% continued medication and 12% (n = 1142) discontinued it. Table 1 shows the baseline characteristics of the discontinuers and continuers, and Table 2 gives the odds ratios (OR) and their 95% confidence intervals (CI) for discontinuation adjusted for the year of statin initiation. Of the demographic factors, only high age was associated with a decreased odds of discontinuation (OR = 0.81; 95% CI, 0.68–0.98), whereas sex, education, and marital status were not. Of the health measures, vascular comorbidity was associated with decreased odds of discontinuation (OR = 0.77; 95% CI, 0.66–0.89), whereas suboptimal perceived health, use of antidepressants, and cancer history were not. Of behavior-related risk factors, overweight (OR = 0.83; 95% CI, 0.71–0.96) and obesity (OR = 0.75; 95% CI, 0.63–0.90) predicted reduced odds of discontinuation among all the participants. Finally, high co-payment (OR = 1.32; 95% CI, 1.05–1.65) predicted increased odds of discontinuation.

In the sex-stratified analyses, age and co-payment were associated with discontinuation among the women only, and the corresponding association of obesity and former smoking was observed among the men only. However, none of these sex differences were significant (test of interaction, all *P* > .15). The only significant difference between the sexes (*P* for interaction = .002) was observed for risky alcohol use: the OR being 0.66 (95% CI, 0.47–0.92) for the men and 1.32 (95% CI, 1.05–1.66) for the women.

Table 3 presents the independent associations of the significant predictors found in Table 2, adjusted for each other, and the year of statin initiation. The results from this fully adjusted model were substantially similar to the model adjusted for the year of statin initiation only. The association of former smoking with discontinuation disappeared for the men in this fully adjusted model (OR = 0.76; 95% CI, 0.56–1.04). The difference between the sexes observed for risky alcohol use was almost unchanged (OR = 0.69; 95% CI, 0.49–0.98 for the men and 1.28; 95% CI, 1.02–1.62 for the women).

## Discussion

### Main findings

In our observational study involving a large cohort of public sector employees, we found that older age, vascular comorbidity, and overweight or obesity were associated with a decreased odds of discontinuation of statin therapy. In contrast, high-patient co-payment of the first statin purchase was associated with an increased odds of discontinuation. Among the women but not in the men, risky alcohol use was additionally associated with an increased risk of discontinuation.

### Comparison with other studies

The rate of discontinuation was 12%, which is within the range reported earlier.^2^, ^3^, ^6^, ^10^, ^11^, ^41^, ^42^ Some previous studies have found that patients with cardiovascular comorbidities are less likely to discontinue statin use than those free of such comorbidities.^3^, ^20^, ^43^ However, a recent Danish, population-based study of 161, 646 new statin users reported discontinuers to have a slightly higher prevalence of almost all the examined diagnoses of comorbidity, including cardiovascular disease.^11^ A major limitation of that study was its reliance on registered data only. As a result, it was not possible to control for co-existing behavior-related risk factors, such as obesity, smoking, and alcohol use, which could have affected the risk of discontinuation. In our study, we were able to control for these health risk behaviors as they appeared before the initiation of statin treatment, and we found that discontinuation was less likely for patients with previous cardiovascular comorbidities than for patients free of them. These findings suggest that patients who are the most likely to benefit from statin therapy are the most likely to continue it. This group of patients may be more motivated than others due to a better understanding of the need for statin treatment.^44^

It is possible that patients discontinue therapy because of poor drug effectiveness or the development of adverse effects. However, in the West of Scotland Coronary Prevention Study, adverse effects accounted for only 2% of discontinuations, with the overall discontinuation rate of 30% at five years.^45^ In a retrospective cohort study of 107,835 patients, more than half of the study patients had their statin discontinued, but only 3.9% of them reported an adverse reaction as the reason for discontinuation.^2^ This potentially unnecessary discontinuation of statins^3^ may lead to preventable cardiovascular events. Indeed, a three times higher risk of myocardial infarction has been found among discontinuers than among patients who continued statin treatment.^46^

In addition to comorbidities, higher age, overweight, and obesity, and, for men, risky alcohol use were significantly associated with a decreased odds of discontinuation. Contrary to these factors, risky alcohol use among the women was associated with an increased rate of statin discontinuation. Previous studies support some of our findings: younger age^11^, ^14^, ^23^, ^47^ and alcohol misuse^48^ have been shown to be associated with an increased risk for statin nonadherence. In addition, obesity in a male population has been shown to decrease the odds of nonadherence (OR = 0.87; 95% CI, 0.81–0.94).^49^

Consistent with previous research, in which lower out-of-pocket expenses had a positive impact on persistence with therapy,^38^ we found that a high level of patient co-payment was an independent factor for increased statin discontinuation. Especially in a high-risk secondary prevention group with a greater total of medications, overall co-payment can be high and thus unnecessary statin-related costs should be avoided.

Discontinuation is an extreme form of nonadherence (which also includes intermittent use of medication). In a previous register study from Finland, half of all statin users discontinued statin for at least 180 days during 10 years of follow-up. Of the discontinuers, 47% restarted statin within one year, and 89% by the end of the follow-up.^50^ In our study, when extending the follow-up period by another year, we found that, of the 1142 participants who discontinued statin medication during the first year of therapy, 18% filled at least one prescription during the second year of statin treatment (i.e., re-entered as a statin user) (data not shown). Thus, it is likely that a substantial proportion of statin discontinuers drop of treatment permanently or for a long period.

### Strengths and limitations of our study

This is a longitudinal study based on register data linked to questionnaires involving demographic characteristics, lifestyle factors, and health status. Our study has a number of strengths. First, it has a large sample size with excellent follow-up. Second, the generalizability of our findings is expected to be greater than in clinical trials as our study involves a large cohort of unselected statin initiators (both men and women) in real-world practice. Third, the Finnish Public Sector cohort contains detailed health status information and lifestyle factors rarely available in prescription claims databases. Finally, owing to the universal drug reimbursement system in Finland and the availability of statins by prescription only, the prescription register provided comprehensive and valid data on statin purchases. All statins (C10AA) were assessed; thus switching to another statin was possible and would not have been wrongly interpreted as discontinuation.

In spite of these strengths, our study also has some limitations. First, we have no information on the reasons for discontinuation of statin therapy or of drug-related adverse effects. Second, we used prescription register information to estimate actual pill intake. This practice meant that primary discontinuers were not included (those who never fill the first prescription). Moreover, as with any pharmacy claim database study, we could only determine that a prescription was filled, not that a patient actually took the medicine.^51^ Third, as we did an all-statin analysis instead of looking at individual statins, we do not know if there are differences in discontinuation between individual statins. Fourth, factors related to the health care system and physician's performances were not available, although they can be associated with discontinuation.^43^, ^52^ Fifth, self-reporting tends to underestimate obesity and overweight^53^ as well as smoking and alcohol use.^54^, ^55^ Finally, our study did not include any measurement of serum lipid levels or an assessment of patients' total cardiovascular risk, which may have affected the perceived need for statin therapy and the discontinuation of it.

## Conclusions and implications for practice and future research

In clinical practice, many patients for whom statins are prescribed discontinue the use of the drug within a year, which is likely to reduce any benefit of medication and increase the risk of cardiovascular events.^56^ Our study suggests that statin discontinuation is common, but several predictors of discontinuation are readily assessable and can provide information with which to identify those with an increased risk of nonadherence. Further intervention studies are needed to assess whether increased efforts to motivate treatment adherence in risk groups would reduce discontinuation and cardiovascular events.

## Figures and Tables

**Table 1:** Characteristics of the 9285 participants who began statin therapy after completing the survey in the Finnish Public Sector Study

Characteristic	All (n = 9285), (%)	Continuer (n = 8143), %	Discontinuer∗ (n = 1142), %
Sex (n = 9285)			
Men	2211 (23.8)	23.7	24.3
Women	7074 (76.2)	76.3	75.7
Age group, y (n = 9285)			
24–50	1971 (21.2)	20.8	24.3
51–60	4811 (51.8)	52.1	49.6
61–75	2503 (27.0)	27.1	26.1
Education (n = 9285)			
High	4363 (47.0)	46.7	49.2
Intermediate	3390 (36.5)	36.9	33.9
Basic	1532 (16.5)	16.4	16.9
Marital status (n = 9144)			
Married	6976 (76.3)	76.3	76.4
Single	2168 (23.7)	23.7	23.6
Co-payment per first package (n = 9285)			
Low (<5 euros)	3700 (39.8)	39.6	41.4
Moderate (5–20 euros)	2642 (28.5)	28.6	27.5
High (>20 euros)	2943 (31.7)	31.8	31.1
Suboptimal self-rated health (n = 9184)			
No	5315 (57.9)	57.7	59.3
Yes	3869 (42.1)	42.3	40.7
Vascular comorbidity† (n = 9285)			
No	6458 (69.6)	68.9	74.4
Yes	2827 (30.4)	31.1	25.6
Cancer (n = 9285)			
No	9110 (98.1)	98.1	98.3
Yes	175 (1.9)	1.9	1.7
Use of antidepressants (n = 9285)			
No	7547 (81.3)	81.1	82.6
Yes	1738 (18.7)	18.9	17.4
Body mass index (n = 9031)			
<25	3286 (36.4)	35.7	41.1
25–29.9	3820 (42.3)	42.7	39.6
≥30	1925 (21.3)	21.6	19.3
Smoking (n = 8822)			
None	3974 (45.0)	44.8	46.7
Former	3327 (37.8)	38.2	34.0
Current	1521 (17.2)	17.0	19.3
Risky alcohol user‡ (n = 9067)			
No	7813 (86.2)	86.3	85.1
Yes	1254 (13.8)	13.7	14.9
Physical activity (n = 9027)			
Active	3159 (35.0)	34.7	37.0
Moderate	3028 (33.5)	34.0	30.5
Low	2840 (31.5)	31.3	32.5

∗ Discontinuer was defined as a person, who filled only one prescription during the first year of statin medication.

† Hypertension, heart failure, coronary artery disease, diabetes, stroke, or arrhythmias.

‡ Risky alcohol user: mean alcohol consumption ≥16 drinks per week for women and ≥24 per week for men or passed out due to heavy alcohol consumption at least once during the 12 months.

**Table 2:** Association between the baseline characteristics and lifestyle factors and statin discontinuation (= filled only one prescription during the first year of statin medication) among the 9285 initiators

Characteristic	All n = 9285	Male n = 2211	Female n = 7074
OR∗ (95% CI)	OR∗ (95% CI)	OR∗ (95% CI)
Sex			
Male (ref)	1.00	na	na
Female	1.01 (0.86–1.17)	na	na
Age group, y			
24–50 (ref)	1.00	1.00	1.00
51–60	0.85 (0.72–1.01)	0.95 (0.70–1.30)	0.80 (0.66–0.97)
61–75	0.81 (0.68–0.98)	1.03 (0.72–1.50)	0.74 (0.59–0.92)
Education			
High (ref)	1.00	1.00	1.00
Intermediate	0.88 (0.76–1.02)	0.85 (0.62–1.17)	0.89 (0.75–1.05)
Basic	1.03 (0.85–1.24)	1.24 (0.87–1.77)	0.96 (0.77–1.20)
Marital status			
Married (ref)	1.00	1.00	1.00
Single	1.00 (0.85–1.16)	0.90 (0.63–1.30)	1.02 (0.86–1.21)
Suboptimal self-rated health			
No (ref)	1.00	1.00	1.00
Yes	0.97 (0.85–1.11)	1.03 (0.79–1.35)	0.95 (0.82–1.11)
Use of antidepressants			
No (ref)	1.00	1.00	1.00
Yes	0.89 (0.75–1.06)	0.96 (0.63–1.46)	0.87 (0.72–1.06)
Cancer			
No (ref)	1.00	1.00	1.00
Yes	0.82 (0.49–1.39)	0.92 (0.32–2.61)	0.79 (0.43–1.45)
Vascular comorbidity†			
No (ref)	1.00	1.00	1.00
Yes	0.77 (0.66–0.89)	0.66 (0.49–0.89)	0.81 (0.68–0.97)
Co-payment per first package			
Low (<5 euros) (ref)	1.00	1.00	1.00
Moderate (5–20 euros)	1.03 (0.87–1.23)	0.87 (0.60–1.24)	1.12 (0.92–1.36)
High (>20 euros)	1.32 (1.05–1.65)	1.02 (0.64–1.60)	1.46 (1.12–1.90)
Body mass index			
<25 (ref)	1.00	1.00	1.00
25–29.9	0.83 (0.71–0.96)	0.80 (0.59–1.07)	0.83 (0.70–0.98)
≥30	0.75 (0.63–0.90)	0.55 (0.36–0.83)	0.83 (0.68–1.01)
Smoking status			
None (ref)	1.00	1.00	1.00
Former	0.88 (0.76–1.02)	0.70 (0.51–0.96)	0.93 (0.79–1.10)
Current	1.09 (0.91–1.31)	0.97 (0.68–1.37)	1.14 (0.92–1.41)
Risky alcohol user‡			
No (ref)	1.00	1.00	1.00
Yes	1.03 (0.85–1.25)	0.66 (0.47–0.92)	1.32 (1.05–1.66)
Physical activity			
Active (ref)	1.00	1.00	1.00
Moderate	0.87 (0.74–1.02)	0.92 (0.66–1.27)	0.85 (0.71–1.02)
Low	0.98 (0.84–1.15)	0.94 (0.68–1.30)	0.99 (0.83–1.19)

CI, confidence interval; OR, odds ratio; ref, reference group; na, not applicable.

∗ Odds ratios adjusted for the year of statin initiation.

† Hypertension, heart failure, coronary artery disease, diabetes, stroke, or arrhythmias.

‡ Risky alcohol user: mean alcohol consumption ≥16 drinks per week for women and ≥24 per week for men or passed out due to heavy alcohol consumption at least once during the 12 months.

**Table 3:** Association between significant characteristics and lifestyle factors and statin discontinuation (= filled only one prescription during the first year of statin medication) among the 9285 initiators. Adjusted for the factors in the model and for the year of statin initiation

Characteristic	All n = 9285	Male n = 2211	Female n = 7074
OR (95% CI)	OR (95% CI)	OR (95% CI)
Age group, y			
24–50 (ref)	1.00	1.00	1.00
51–60	0.86 (0.73–1.01)	0.96 (0.70–1.31)	0.82 (0.68–1.00)
61–75	0.82 (0.68–0.99)	1.02 (0.70–1.48)	0.76 (0.61–0.95)
Vascular comorbidity∗			
No (ref)	1.00	1.00	1.00
Yes	0.80 (0.68–0.93)	0.70 (0.52–0.95)	0.83 (0.70–0.99)
Co-payment per first package			
Low (<5 euros) (ref)	1.00	1.00	1.00
Moderate (5–20 euros)	1.04 (0.88–1.23)	0.86 (0.60–1.23)	1.12 (0.92–1.36)
High (>20 euros)	1.29 (1.0–31.62)	0.99 (0.63–1.55)	1.41 (1.09–1.83)
Body mass index			
<25 (ref)	1.00	1.00	1.00
25–29.9	0.85 (0.73–0.98)	0.86 (0.64–1.16)	0.85 (0.72–1.01)
≥30	0.82 (0.69–0.99)	0.65 (0.43–0.99)	0.88 (0.71–1.08)
Smoking status			
None (ref)	1.00	1.00	1.00
Former	0.87 (0.75–1.01)	0.76 (0.56–1.04)	0.90 (0.76–1.06)
Current	1.08 (0.90–1.29)	1.06 (0.75–1.50)	1.05 (0.85–1.30)
Risky alcohol user†			
No (ref)	1.00	1.00	1.00
Yes	1.03 (0.85–1.25)	0.69 (0.49–0.98)	1.28 (1.02–1.62)

CI, confidence interval; OR, odds ratio; ref, reference group.

∗ Hypertension, heart failure, coronary artery disease, diabetes, stroke, or arrhythmias.

† Risky alcohol user: mean alcohol consumption ≥16 drinks per week for women and ≥24 per week for men or passed out due to heavy alcohol consumption at least once during the 12 months.
